# Characteristics of HLA-E Restricted T-Cell Responses and Their Role in Infectious Diseases

**DOI:** 10.1155/2016/2695396

**Published:** 2016-09-06

**Authors:** Simone A. Joosten, Lucy C. Sullivan, Tom H. M. Ottenhoff

**Affiliations:** ^1^Department of Infectious Diseases, Leiden University Medical Center, 2333 ZA Leiden, Netherlands; ^2^Department of Microbiology and Immunology, The University of Melbourne, Peter Doherty Institute for Infection and Immunity, Melbourne, VIC 3010, Australia

## Abstract

Human HLA-E can, in addition to self-antigens, also present pathogen-derived sequences, which elicit specific T-cell responses. T-cells recognize their antigen presented by HLA-E highly specifically and have unique functional and phenotypical properties. Pathogen specific HLA-E restricted CD8^+^ T-cells are an interesting new player in the field of immunology. Future work should address their exact roles and relative contributions in the immune response against infectious diseases.

## 1. Introduction 

T-cell activation requires specific recognition of antigen presented as small fragments (peptides) bound to major histocompatibility complex (MHC) molecules. The recognition of a particular peptide-MHC occurs through a highly specific T-cell receptor (TCR), which is selected in the thymus. Following TCR triggering, costimulation and the presence of polarizing cytokines together determine the T-cell activation pattern and guide ultimate T-cell differentiation. Classically, CD4^+^T-cells recognize antigens scavenged extracellularly by the antigen presenting cell (APC) that are presented in MHC class II, whereas CD8^+^ T-cells recognize endogenous antigens presented by MHC class I (MHC-I) [1, 2]. In spite of this widely held view, already decades ago it was shown that also antigens derived from intracellular pathogens such as viruses or intracellular bacteria can be presented in MHC-I [3]. More recently, cross-presentation by dendritic cells and autophagy have been elucidated as important mechanisms in this context [2, 4].

Transplantation of hematopoietic cells as well as solid organs and detailed studies of viral infections provided the initial key information leading to the concept of genetic MHC restriction by autologous MHC molecules. This is currently often referred to as “conventional” or “donor-restricted” immunity [5]. However, numerous T-cell subsets have been identified that do not fulfil these criteria, including MHC class Ib restricted T-cells, CD1 restricted T-cells, MR1 restricted mucosal associated invariant T-cells (MAIT), NKT-cells, and *γδ* T-cells, subsets that are collectively called “unconventional” or “donor-unrestricted T-cells” (DURT) [5]. Unconventional T-cells behave differently in terms of memory, kinetics, and ligands recognized compared to conventional T-cells as recently summarized [5].

An intriguing group of DURT family cells are the T-cells that are restricted by MHC class Ib molecules. These cells may share several critical properties with conventional T-cells but most importantly recognize antigens typically in the context of nonpolymorphic MHC-I molecules. The human MHC class Ib family, also called nonclassical HLA class I, is comprised of HLA-E, HLA-F, and HLA-G. The major difference with classical class Ia molecules is their very low level of allelic variation. Whereas HLA class Ia families are composed of several hundred family members for HLA-A, HLA-B, and HLA-C alleles, HLA-E, HLA-F, and HLA-G comprise only 3, 4, and 10 family members, respectively, and not all of these are actually expressed as functional proteins [6]. Immune cells express relatively high levels of HLA-E protein, but also tissue cells can express the HLA-E protein (http://www.proteinatlas.org/). Although HLA-E was originally described to be broadly expressed by almost all cells that also express HLA class Ia molecules [7], other studies suggest HLA-E expression is restricted to lymphoid and endothelial cells [8]. Furthermore, pathogens can affect HLA-E cell surface expression; for example, human cytomegalovirus (CMV) can upregulate its expression [9]. HLA-E functions as ligand for CD94-NKG2 receptors and has a peptide-binding groove that is ideally suited for binding peptides derived from the leader sequences of other MHC-I molecules [10]. In this regard, the loss of leader-peptide loaded HLA-E expression is a marker for cells having lost expression of HLA class Ia molecules, which targets these cells for recognition and lysis by Natural Killer (NK) cells [10]. In contrast to HLA-E, HLA-F expression appears to be more restricted and is detected mostly in liver and bladder [10]. However, its expression is largely intracellular and in association with other MHC-I molecules, which has led to speculations that HLA-F might be involved in the intracellular stabilization of HLA class Ia molecules [10]. The third human MHC class Ib family member, HLA-G, has an even more narrow tissue distribution; its expression appears limited to trophoblasts in the placenta, and it has been associated with fetal-maternal tolerance [10]. HLA-G may function during pregnancy to inhibit NK mediated lysis as trophoblasts lack HLA-A and HLA-B expression [11].

Thus, given the intracellular expression of HLA-F and the placental restriction of HLA-G, limited information is available on T-cells interacting with these molecules, and their relevance to general immunity remains unclear. For this reason, the focus of this review will be on HLA-E restricted T-cells.

## 2. HLA-E

**Figure 1:**
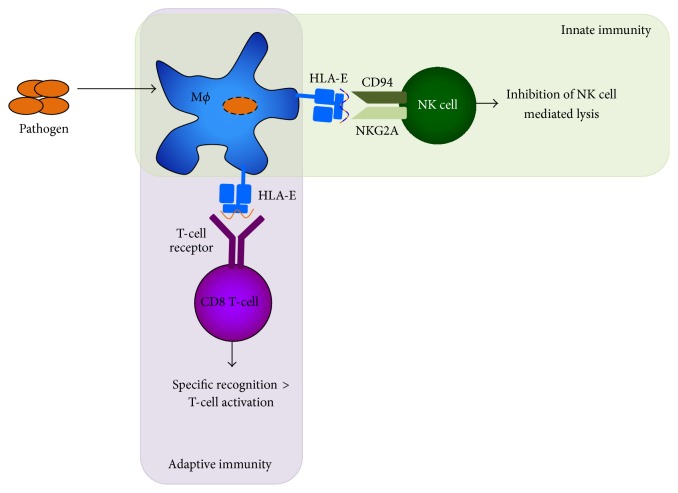
HLA-E serves a dual role in the immune system. HLA-E presents antigens, including pathogen-derived antigens on the cell surface of most cells. NK cells, as part of the innate immune system, will sense the presence of HLA-E presenting self or pathogen-derived peptides and thereby receive inhibitory signals from the CD94/NKG2A complex such that NK mediated lysis will be inhibited. In addition, CD8^+^ T-cells may specifically recognize foreign peptide presented by HLA-E and become activated through their T-cell receptor, resulting in T-cell activation, expansion, and memory formation in the adaptive immune system.

The role of HLA-E in the innate immune response is to present signal sequence-derived peptides of other HLA class I molecules to inhibit NK mediated lysis of cells via recognition by CD94/NKG2A [13]. However, HLA-E can also bind and present other peptide sequences, which can be self or pathogen derived and can be recognized by adaptive T-cells. HLA-E is thus considered to play a role in both innate and adaptive immunity, via interacting with both NK cells as well as presenting peptides to antigen specific CD8^+^ T-cells (Figure 1).

Eleven alleles have been reported for HLA-E, only 3 of which can be translated into proteins, 2 of them being highly dominant, the HLA-E^R^ (E^*∗*^01:01) and the HLA-E^G^ (E^*∗*^01:03) variants, which differ only in a single amino acid at position 107, being arginine (E^*∗*^01:01) or glycine (E^*∗*^01:03). Position 107 is located on the loop between the *β*-strands outside of the *α*2 domain of the heavy chain, just outside the peptide-binding groove. The frequency of the HLA-E^R^ and HLA-E^G^ in the population is about equal, suggesting balanced selection in diverse populations [14, 15]. Whether HLA-E^R^ and HLA-E^G^ display functional differences has not been studied in detail [14, 16], but it has been demonstrated that HLA-E^G^ homozygous cells express higher levels of HLA-E and had higher peptide-binding affinity [16]. More recently, peptide elution studies revealed a different peptide-binding repertoire eluted from HLA-E^R^ versus HLA-E^G^ molecules, indicating that the F-pocket of HLA-E^G^ bound a smaller variety of peptides and had a stronger preference for a lysine at the pΩ position [17, 18].

### 2.1. HLA-E Peptide Binding

The structural basis of HLA-E's ability to bind signal sequence-derived peptides from HLA class Ia (HLA-I) molecules has been studied previously. Unlike HLA class Ia that typically contain 2 or 3 anchor residues, HLA-E contains 5 anchor residues in the peptide-binding groove that highly constrains the sequence of the bound peptide [19, 20]. However, HLA-E was additionally shown to bind peptides derived from viruses such as influenza M1 protein and EBV BZLF1 [21]. Moreover, a sequence from cytomegalovirus (CMV) glycoprotein UL40, which is identical to the HLA-C^*∗*^03 leader sequence, can bind to HLA-E and is capable of preventing NK mediated lysis [9, 22]. The same was found for a HCV derived sequence, despite its sequence difference from signal peptides [23]. Peptide identification and characterization using random peptide approaches also revealed that a leucine on P9 is a critical anchor residue for HLA-E binding but did not identify methionine as critical P2 anchor for HLA-E binding and folding [24]. Identification of the motif within the HLA class Ia leader sequences critical for interaction with the CD94/NKG2 complex suggested anchor residues at positions 2, 6, 7, and 9, while solvent exposed residues at P5 and P8 were likely important for binding to CD94/NKG2 [25]. Thus, while P2 and P9 are anchor residues for binding to HLA-E and P5 and P8 are involved in the interaction with CD94/NKG2 [26], P8 is also the critical residue distinguishing self (signal sequence) from nonself (CMV UL40) and allowing for TCR recognition of CMV UL40 [12].

Recent peptide elution studies provided important insights in the types of peptides that are naturally presented by HLA-E and identified a larger array of peptides eluted from HLA-E than originally discovered [17, 18, 27]. Eluted peptides were generally short (8-9-10 mers), but occasionally also longer peptides were eluted including 11–17 mers [17, 18, 27]. This is in line with earlier studies, where also peptides greater than 8 amino acids could bind HLA-E [21]. Furthermore, eluted peptides were different from signal sequences, possessing hydrophobic amino acids on P2 and P9, consistent with a binding motif that was very similar to that of HLA-A2 [27]. Recent studies in rhesus macaques immunized with SIV-gag (Simian Immunodeficiency Virus), in specific CMV vectors, revealed a series of peptides that were recognized by CD8^+^ T-cells but only a minority contained the canonical MHC class I antigen E (MHC-E) binding motif [28]. Structural analyses revealed that the peptide-binding cleft of HLA-E is rigid but relatively open compared to that of HLA class Ia family members. Peptides that lack the canonical residues can adopt a backbone structure that is similar to canonical peptides, allowing them to bind the HLA-E molecule [28]. These unique binding properties of HLA-E may explain the observed epitope diversity and breadth in SIV-gag in the rhesus macaque vaccination studies [28]. Furthermore, the authors suggested that the open structure of HLA-E may allow peptide exchange [28]; this may be in particular relevant for* Mycobacterium tuberculosis *(Mtb) as HLA-E expression is enriched in the Mtb phagosome [29]. Intriguingly, we have identified a large series of Mtb epitopes presented in HLA-E and recognized by mycobacteria exposed human donors, many of which lack the canonical residues [30].

Together, these data indicate that HLA-E binds signal sequence-derived peptides not only from MHC class Ia molecules but also from other self and even pathogen-derived sequences. Although many peptides contain canonical amino acids for binding HLA-E, clear examples exist for peptides which lack canonical residues and can still bind HLA-E. Presentation of nonself sequences, being absent during thymic selection, may elicit adaptive immune responses by CD8^+^ T-cells.

### 2.2. HLA-E Restricted T-Cells

Specific recognition of pathogen-derived sequences presented by the unconventional presentation molecule HLA-E by the CD8^+^ TCR could lead to specific activation of adaptive immune responses, independent of classically HLA restricted CD4^+^ and CD8^+^ T-cells. In several infectious disease models, evidence for such responses has been reported recently.

#### 2.2.1. Viral Antigens

As viruses require the human host to survive and therefore reside within host cells, their proteins are presented by HLA class I molecules, including HLA class Ib. Peptides from* Epstein Barr Virus *(EBV) [34–36],* Cytomegalovirus *(CMV) [12, 37–41], and* Hepatitis C Virus* (HCV) [44] can be presented by HLA-E and are recognized by virus specific T-cells (Table 1). The peptide epitopes studied from CMV UL40 are highly similar to the HLA class Ia signal sequences, whereas the sequences from EBV BZLF1 and HCV appear more different. The functional and phenotypical description of these T-cells is rather limited, but they all express CD8^+^ as expected for HLA class I restricted cells. In many studies, HLA-E restricted T-cells have been identified and enumerated using HLA-E tetramers whereas functional analyses were limited to the demonstration of target cell lysis. Likewise, phenotypical characterizations were very limited in scope but when performed showed a cytolytic T-cell phenotype (perforin, granzyme A/B) and IFN*γ* production in some studies [38, 41].

(*1) HLA-E and CMV*. Initial studies on possible recognition of HLA-E peptide complexes by T-cells in a TCR dependent manner were performed using signal sequences from HLA class Ia alleles [34, 35, 42] (Table 1). It is* a priori* not clear why healthy human subjects would mount T-cell responses towards signal sequences of conserved class Ia molecules. While some of these studies lack information on the HLA-typing of the donors, others have suggested that these cells are mostly reactivity against nonself target peptides of signal sequences [34, 35, 42]. Similarly, cytotoxic CD8^+^ T-cells recognizing HLA-E binding sequences from TCR V*β* chains were detected in peripheral blood [33]. An alternative, nonexclusive, explanation may be that these T-cells are reactive with nonself virally derived antigens that share sequence homology with the MHC class Ia derived signal sequences used in these studies.

Involvement of the TCR in specific recognition of HLA-E was derived from “NK-CTL” clones. Although these T-cells were originally termed “NK-CTL” due to their ability to lyse a broad range of allogeneic targets, it was subsequently found that these T-cells were specific for the CMV UL40-encoded peptide (VMAPRTLIL) bound to HLA-E [35, 42]. The detected alloreactivity was due to target cells possessing HLA-C alleles encoding the same sequence as the UL40 peptide (e.g., HLA-C^*∗*^03). UL40-specific T-cells can reach frequencies in the circulation similar to those restricted by classical HLA-I, indicating their potential to play a significant role in CMV immunity [50].

**Figure 2:**
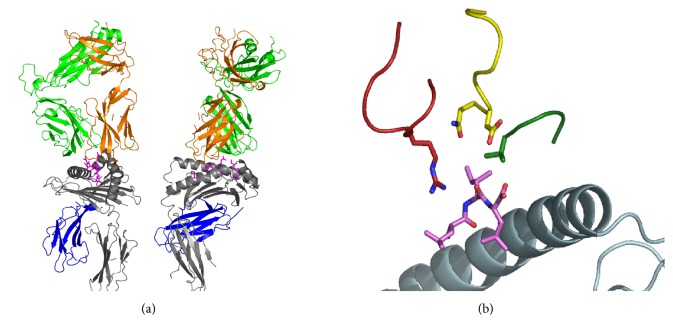
Crystal structure of the HLA-E/KK50.4 TCR complex [12]. (a) Overview of the HLA-E/KK50.4 TCR structure showing two orthogonal views. The HLA-E heavy chain is shown in grey ribbons, the UL40 peptide in pink sticks, and *β*2m in blue ribbons. The KK50.4 alpha and beta chains are shown as orange and green ribbons, respectively. (b) The convergence of all 3 CDR *β* loops onto P8 Ile of the peptide bound to HLA-E. The HLA-E heavy chain is grey, peptide is pink, CDR1*β* is yellow, CDR2*β* is green, and CDR3*β* is shown in red.

The TCR from an UL40-specific T-cell clone, KK50.4, was cloned, expressed, and analysed for its interaction with HLA-E in complex with the UL40-epitope VMAPRTLIL (Figure 2) [12]. Overall, the structural basis for recognition of HLA-E largely overlaps that of TCR recognition of HLA class Ia. However, in order for UL40-specific T-cells to recognize the UL40 antigen or allogeneic HLA-I peptides, the UL40-specific TCR need to distinguish between the UL40 epitope (VMAPRTLIL) and nearly identical self peptides which may differ by as little as a single methyl group (e.g., VMAPRTLVL). Analysis of TCR sequences from UL40 specific T-cell clones suggested there were a limited number of TCRs capable of such discrimination, as all of the clones isolated utilized TRBV14 (V*β*16) and there was a characteristic arginine residue present in the CDR3*β* (Figure 2) [12]. Structural analyses of the KK50.4 clone showed that the convergence of CDR1, CDR2, and CDR3 of the KK50.4 *β* chain onto P8 Ile determined self/nonself discrimination (Figure 2). Notably, the highly selected arginine present in the CDR3*β* made multiple contacts with both HLA-E and peptide.

As the CMV UL40 peptide is highly homologous to HLA class Ia derived signal sequences, typically these T-cells are only observed in individuals where the UL40 epitope differs from that found in self-HLA-C alleles (e.g., HLA-C^*∗*^07 homozygotes) [12]. However, other pathogen-derived peptides that bind to HLA-E are more different from the class Ia signal sequences and may therefore depend less on the donors' HLA class Ia genotype.

(*2) HLA-E and HIV*. HIV-nef proteins interact with the intracellular domain of HLA-A and HLA-B molecules, resulting in downregulation of HLA class Ia molecules from the cell surface. In contrast, HLA-C and HLA class Ib molecules, particularly, HLA-E and HLA-G, lack these intracellular nef-interaction domains and thus remain expressed normally on the cell surface of HIV infected cells [51]. In addition, it has been shown that a peptide from the HIV-1 capsid protein p24 (AISPRTLNA) may further enhance HLA-E surface expression [52]. However, recently it was shown that this peptide presented in HLA-E is not recognized by CD94/NKG2A and that these cells thus were not protected against NK mediated T-cell lysis [53]. This is most likely due to lack of homology between the HIV p24 peptide and HLA leader peptides, which prevents ligation of CD94-NKG2A to the HLA-E peptide complex [53]. Inhibition of NK mediated lysis of HIV-1 infected T-cells rather appears to be the result of HLA-C expression and recognition by NK cells that specifically express the NK cell receptors KIR2DL1/2/3 [53]. Interestingly, it has not yet been investigated whether the HIV-1 p24 peptide, presented in HLA-E, may be recognized by the host adaptive immune system and thus may result in specific CD8^+^ T-cells. Induction of such CD8^+^ T-cell responses would be interesting from a therapeutic as well as vaccination point of view.

In rhesus macaques, MHC-E (or Mamu-E) is the homologue of human HLA-E, which also showed upregulated expression in HIV/SIV infected animals [28]. Vaccination of rhesus macaques with a CMV-based vector, expressing HIV gag, revealed a very strong CD8^+^ T-cell response (*αβ* TCR) with a large variety of specific interactions, with an estimated induction of 4 distinct epitopes per 100 amino acids in all tested HIV/SIV derived antigens [28]. Although this massive MHC-E restricted response was due to the specific design of the viral vector, it clearly illustrates the abundance of potential HLA-E epitopes in a large array of antigens. Detailed characterization of the peptide-binding domain revealed a relatively open structure, exposing many side chains for interaction with the TCR [28]. Interestingly, next to inducing MHC-E restricted T-cells, these CMV recombinant vectors also induced a significant population of other unconventional CD8^+^ T-cells, which were restricted by MHC class II molecules.

#### 2.2.2. Bacterial Antigens

Bacteria like* Salmonella* and Mtb are intracellular pathogens that hijack host cells to promote their own survival. Intriguingly, the expression of HLA-E is enriched on Mtb phagosomes compared to classical HLA class Ia family members, thus presumably facilitating HLA-E loading by Mtb peptides in infected cells [29]. In 1998, Lewinsohn et al. identified Mtb specific CD8^+^ T-cell clones that appeared to be restricted to MHC class Ib [31], two of which were HLA-E restricted [32]. However the Mtb derived peptide ligands were not identified. Unpublished recent data point to a peptide derived from the Mtb glycoprotein Mpt32 (David Lewinsohn, personal communication, manuscript in preparation). In an independent effort, we have screened the Mtb genome for the presence of peptides that could potentially be presented by HLA-E and selected 69 peptides based on 3 different prediction algorithms [30]. Many of these peptides were recognized by donors that had been previously sensitized by mycobacteria, suggesting* in vivo *priming and T-cell memory for several HLA-E epitopes [30]. We have shown that these peptides presented in HLA-E elicit CD8^+^ T-cell activation through the TCR, as measured by both ZAP70 phosphorylation (the first downstream effect in TCR signalling), as well as CD137 expression (a molecule exclusively expressed following specific antigen recognition on CD8^+^ T-cells) [47]. Moreover, HLA-E restricted T-cell lines specific for Mtb had strongly reduced cytokine production in the presence of blocking antibodies against the *αβ*TCR or HLA-E [48]. Altogether, these data support specific recognition of HLA-E peptide complexes by Mtb specific TCRs. HLA-E binding peptides from Mtb were not capable of preventing NK mediated lysis in a CD94/NKG2A dependent manner [48], similar to HIV p24 [53], indicating that only surface expression of peptide containing HLA-E may not be sufficient.

Detailed characterization of these T-cells revealed that they are cytolytic or suppressive, and that T-cells reactive against the same peptide can display different functional polarities, indicating that polarity is not determined by the peptide. Interestingly, T-cell clones with cytolytic activity were also capable of inhibiting intracellular outgrowth of Mtb, suggesting that they are potent antimycobacterial effector cells [47]. Many of the HLA-E restricted Mtb specific T-cells did not produce typical cytotoxic T-lymphocyte associated cytokines, nor did they produce classical Th1 cytokines (IFN*γ*, TNF, and IL2), but instead they produced an array of Th2 cytokines including IL-4, IL-5, IL-10, and IL-13, as well as the Th2 associated transcription factor GATA-3 (Table 2) [47, 48]. In patients with TB disease, HLA-E tetramers identified Mtb specific HLA-E restricted CD8^+^ T-cells, with the highest frequencies at TB diagnosis and waning of the response during successful treatment [48]. Moreover, in line with the knowledge that HLA-E is not susceptible to downregulation by HIV, we were able to detect HLA-E specific T-cells in patients that concomitantly were infected with Mtb and HIV [48].

Salmonella peptides presented by HLA-E are also recognized by HLA-E restricted T-cells. Volunteers vaccinated with a* S. typhi* vaccine had a robust HLA-E restricted T-cell response, as measured by the cytolytic capacities of these cells, such as granzyme B activity (Table 1) [43]. Kinetic analysis of these responses in a similarly vaccinated cohort revealed that the HLA-E restricted T-cells are long-lasting, up to 2 years after vaccination, again suggesting immune memory [46]. Moreover, following challenge experiments with* Salmonella* in unvaccinated, healthy volunteers, multifunctional HLA-E restricted CD8^+^ T-cells were detected and correlated with protection against typhoid disease development [49].

#### 2.2.3. Tumor and Self-Antigens

Interestingly, T-cells reactive with self Hsp60sp presented by HLA-E have also been identified, both in healthy donors and in patients with type 1 diabetes [45]. These CD8^+^ T-cell lines were involved in discriminating self from nonself in the periphery, and defective discrimination between self and nonself was detected in the majority of patients with type 1 diabetes [45]. While many viruses are known to interfere with antigen processing and presentation, resulting in peptide presentation in a TAP-independent manner, this is also the case in many tumors. As a consequence, tumor unique antigens may be presented also in the context of HLA-E [54, 55], which may subsequently be recognized by cytotoxic T-cells [55]. In contrast to what was expected, in humans the presence of CTLs was only beneficial in patients with lung carcinoma if HLA-E was not expressed by the tumor, indicating that HLA-E restricted CTLs may not directly contribute to tumor elimination in these patients [56].

#### 2.2.4. Autoimmune Diseases

HLA-E restricted T-cell responses have been studied only to a very limited extent in autoimmune diseases; however they could potentially play an important role. In patients with multiple sclerosis (MS), increased frequencies of EBV specific, HLA-E restricted CD8^+^ T-cells have been found to be associated mostly with the relapsing remitting form of the disease rather than the progressive form [36]. Moreover, CD8^+^ T-cells induced by glatiramer acetate vaccination appear to be restricted to HLA-E and have immunomodulatory capacities, resulting in amelioration of MS [57–59]. HLA-E restricted CD8^+^ T-cells in patients with MS appeared phenotypically different from healthy controls; however these T-cells were selected based on the expression of NKG2C and thus may reflect only a small subset of HLA-E restricted T-cells [60]. In rheumatoid arthritis (RA), limited information is available, although HLA-E polymorphisms may be associated with disease susceptibility and treatment responsiveness [61]. Interestingly and similar to the studies in MS, RA like autoimmunity can be strongly inhibited by induction of (self Hsp60) peptide specific Qa-1 restricted suppressor T-cells in mice [62]. Furthermore, there is a defect in CD8^+^ T-cell recognition of HLA-E/Hsp60sp in patients with type I diabetes [45]. Thus, MHC-E restricted CD8^+^ T-cells with immunoregulatory properties may be critical in amelioration of autoimmune diseases and deserve further detailed characterization.

### 2.3. Properties of HLA-E Restricted Human T-Cells

Surprisingly, little information is available on the phenotype and function of human CD8^+^T-cells recognizing peptides presented by HLA-E. In many studies, HLA-E restricted T-cells have only been enumerated using tetramer staining, or the presence of HLA-E reactivity was demonstrated using cytolytic assays (Table 2). Analyses of the TCR composition were only performed in a limited number of studies and, as mentioned above, found consistent selection of TRAV14 (V*β*16) in CMV specific TCRs [12]. Additional studies have also determined V*β*22 usage in CMV restricted TCRs [41].

Basic descriptive information of HLA-E restricted T-cells, such as memory phenotype, is also largely lacking. The limited data that have been published do not suggest specific memory stages to be overrepresented among HLA-E restricted CD8^+^T-cell populations, since both CD45RA positive and negative populations were identified, as was also reported for CCR7 (Table 2). Generally, the T-cells reported expressed cytolytic molecules, but in some cases these were weakly expressed or even undetectable (Table 2). Specific target cell lysis was frequently used as read out to demonstrate HLA-E restriction of CD8^+^ T-cells. Recently, we demonstrated for the first time that HLA-E restricted CD8^+^ T-cell clones had antibacterial activity against Mtb, considered to be an important property in immune control of intracellular pathogens (Table 2). Other studies have not assessed or reported viral or bacterial inhibition following HLA-E restricted CD8^+^ T-cell activation or downstream target cell lysis. It will be of interest to identify the mechanism of control of intracellular outgrowth of Mtb, as this could be a yet unknown component of the immune system that could be harnessed for preventive or therapeutic interventions.

Cytokine production has been analysed in detail only in the most recent series of papers. Originally, studies focused on the production of classical Th1 cytokines, such as IFN*γ* and TNF*α*, and although sometimes detected in HLA-E restricted T-cells, not all produced IFN*γ* in response to specific peptide stimulation (Table 2). As mentioned above, we recently found Mtb specific HLA-E restricted CD8^+^ T-cells to produce Th2 rather than Th1 cytokines and demonstrated that these cells utilize IL-4 to activate B-cells (Table 2). It would be interesting and relevant to investigate Th2 cytokine production also in HLA-E restricted CD8^+^ T-cells in response to other ligands. Moreover, an analysis of transcription factor expression in HLA-E restricted CD8^+^ T-cells has been limited thus far to our description of Mtb specific T-cells expressing GATA-3 (Table 2).

One of the major differences between innate and adaptive immunity is the formation of immunological memory during adaptive immune responses. As HLA-E restricted CD8^+^ T-cells are activated through their TCR, in an antigen specific manner, it is likely that they also differentiate into memory cells. The formation of memory cells following HLA-E mediated antigen presentation would be an important prerequisite for successful application of these peptides in future vaccination strategies. However, information on T-cell memory development is limited. Phenotypically, effector memory [30] and effector memory recently activated [38, 41] have been described as indicators of memory. Moreover, screening for recognition of HLA-E restricted Mtb peptides by human donors showed recognition only in donors that had been sensitized by mycobacteria (as measured by PPD recognition), suggesting that* in vivo* priming, and thus memory induction, was critical [30]. Vaccination of healthy volunteers with a single dose of* S. typhi* strain Ty21a resulted in antigen specific, HLA-E restricted CD8^+^ T-cells that were detectable up to 2 years after vaccination, suggesting that HLA-E restricted memory T-cells had been induced [46]. Also, individuals with latent TB and individuals successfully treated for TB disease still had circulating CD8^+^ T-cells binding to HLA-E tetramers (TM) loaded with Mtb peptides (notwithstanding that the frequencies detected were highest in patients with active TB disease); the persistence of responses after microbiological cure thus also suggests immune memory [48].

### 2.4. Qa-1^b^: The Murine HLA-E Homologue

The murine homologue of human HLA-E is Qa-1^b^, which also presents signal sequences from MHC class I proteins that are called Qdm (Qa-1 determinant modifier) [54]. Qa-1^b^ can also bind CD94/NKG2A complexes to silence NK cell activity. However, also specific recognition of Qa-1^b^ peptide complexes by T-cells has been described [54]. Interestingly, similar to HLA-E, Qa-1^b^ restricted T-cells have been isolated that are able to detect differences in leader-sequenced derived peptides between mouse strains [63], although there is no indication they are involved in immunity to mouse CMV. Also, similar to HLA-E, Qa-1^b^ restricted T-cells have been implicated in immunity to pathogens, including* Listeria monocytogenes* [64] and* Salmonella typhimurium* [65].

Qa-1^b^ restricted* S. typhimurium *specific T-cells are curiously cross-reactive to self Hsp-60 derived peptides and thus have been implicated in autoimmune conditions [66]. Furthermore, there is a large and older body of research focusing on Qa-1^b^ restricted suppressor CD8^+^ T-cells. These T-cells reportedly recognize Qa-1^b^ in a TCR dependent manner and act to suppress autoreactive CD4^+^ T-cells, thereby attenuating the development of autoimmune encephalomyelitis (a mouse model of multiple sclerosis), very similar to the role of HLA-E restricted CD8^+^ T-cells evoked by vaccination in human MS [67–69]. In this model, Qa-1^b^ deficient mice developed exaggerated secondary CD4 responses, as a result of the lack of a population of regulatory CD8^+^ T-cells, demonstrating the* in vivo* significance of suppressor Qa-1^b^ restricted T-cells. Qa-1^b^ restricted T-cells appear critical for the maintenance of self-tolerance. Their role in infectious diseases and in particular in the elimination of pathogens has not been studied in great detail thus far. Moreover, a detailed description of the phenotype and function of these cells is lacking and warrants further investigation.

## 3. Summary and Implications

HLA-E plays a dual role in the innate and adaptive immune system (Figure 1). The low polymorphism in HLA-E in conjunction with its relative insensitivity to downregulation by, for example, HIV, makes HLA-E an interesting target for vaccination strategies against infectious diseases and tumors. Small sets of peptides should suffice to induce T-cells recognizing foreign antigens and mount effector responses. The quality of these T-cell responses needs further investigation, however, given the diversity in functions that have been described thus far, ranging from cytotoxicity and pathogen control to immune suppression. Various pathogen-derived antigens can bind and stabilize HLA-E at the cell surface, for some of which this may be an essential mechanism to prevent NK mediated target cell lysis. However, many of these antigens can also be recognized in a TCR dependent manner by CD8^+^ T-cells. These T-cells may suppress bystander T-cells or lyse (infected) target cells and inhibit intracellular bacteria, indicating an important functional contribution to the immune response. Nevertheless, the relative frequency of HLA-E restricted T-cells and their* in vivo *relevance in many cases remains unknown and unstudied. Although these T-cells are donor-unrestricted, they in many aspects display similar functionalities to classical, conventional T-cells.

Pathogen specific HLA-E restricted CD8^+^ T-cells are an interesting new player in the field of immunology. Future work should address their exact roles and relative contributions in the immune response against infectious diseases.

## Figures and Tables

**Table 1:** Antigen types/characteristics presented in HLA-E and recognized by T-cells.

Sequence	Origin	Description of antigen	Patients/donors	Recognition by T-cells	Reference
?	*Mycobacterium tuberculosis*	Glycopeptide from Mpt32^*∗∗*^	T-cell clones derived from latently infected individuals	IFN*γ* production	[31, 32]

SLELGDSAL	Human	TCR V*β*1	Healthy donors, *in vitro* priming of CD8^+^ T-cells	Target cell lysis	[33]
LLLGPGSGL		TCR V*β*2			

SQAPLPCVL	*Epstein Barr Virus*	BZLF1	T-cells from healthy donor; MS patients	T-cell proliferation, target cell lysis, TM staining	[34–36]

VTAPRTVLL	Human	HLA-B15	T-cell lines from healthy donors	T-cell proliferation, target cell lysis	[34]
VMAPRALLL	Human	HLA-Cw7			
VMAPRTLFL	Human	HLA-G1			

VMAPRTLIL	*Cytomegalovirus*	UL40	T-cell lines from healthy donors; lung transplant recipients	NK-CTL proliferation, target cell lysis, TM staining	[12, 34, 35, 37–42]
VMAPRTLVL					
VMAPRTLLL					

GMQFDRGYL	*Salmonella enterica serovar typhi*	*S. typhimurium* GroEL	Healthy volunteers vaccinated with *S. typhi* strain Ty21a	Target cell lysis	[43]
AMLQDIATL					
KMLRGVNVL					
VEGEALATL					
AAVEELKAL					
AVAKAGKPL					
KLQERVAKL					

YLLPRRGPRL	*Hepatitis C Virus*	Peptide HCV core aa 35–44	Chronic hepatitis C patients	IFN*γ* production	[44]

69 peptides	*Mycobacterium tuberculosis*	Multiple proteins expressed by Mtb	Latently infected individuals, BCG vaccinated newborns	CD8^+^ T-cell proliferation	[30]

QMRPVSRVL	Human	Hsp60sp	Healthy donors, patients with type I diabetes	T-cell proliferation, target cell lysis	[45]

?	*Salmonella enterica serovar typhi*	?	Healthy volunteers vaccinated with *S. typhi* strain Ty21a	Cytokine producing T-cells	[46]

RMPPLGHEL	*Mycobacterium tuberculosis*	Rv2997, alanine rich dehydrogenase	T-cell clones derived from latently infected individuals	CD137 expression, ZAP70 phosphorylation; CD8^+^ T-cell proliferation, TNF*α* production, TM staining	[47, 48]
VLRPGGHFL	*Mycobacterium tuberculosis*	Rv1523, methyltransferase			

VMTTVLATL	*Mycobacterium tuberculosis*	Rv1734c, conserved hypothetical protein	Patients with pulmonary TB	CD8^+^ T-cell proliferation, TNF*α* production, TM staining	[48]
RLPAKAPLL	*Mycobacterium tuberculosis*	Rv1484, NADH-dependent enoyl reductase InhA			
VMATRRNVL	*Mycobacterium tuberculosis*	Rv1518, unknown, possibly glycosyl transferase			
VLRPGGHFL	*Mycobacterium tuberculosis*	Rv1523, methyltransferase			

?	*Salmonella typhi*	?	Healthy donors challenged with *Salmonella typhi*	Cytokine production	[49]

^*∗∗*^D. Lewinsohn, personal communication, manuscript in preparation.

**Table 2:** T-cell properties of MHC class Ib restricted T-cells; phenotype, cytokine production, transcription factors, cytolytic molecules.

	Antigen	T-cell phenotype																								Cytokines										Transcr. factors					Function				Reference
		CD3	CD8	TCR*αβ*	CD25	LAG3	CD39	CTLA4	CD45RA	CD45RO	CCR7	CD27	CD28	CD56	CD57	CD62L	CD94	Perforin	Granzyme A	Granzyme B	Granulysin	CD107a	TRAIL	TCR V*β*16	TCR V*β*22	IFN*γ*	TNF*α*	IL-2	IL-4	IL-5	IL-10	IL-13	IL-17	TGF-*β*	MIP-1*β*	T-bet	GATA3	RORC	FOXP3	EOMES	Spec. target lysis	Path. killing	T-cell suppr.	TM binding	
Self	VMAPRTLVL		+	+																																					+				[42]

Self	VTAPRTVLL		+																					+																	+				[34]
	VMAPRTLVL		+																					+																	+				
	VMAPRTLIL		+																					+																	+				
	VMAPRALLL		+																					+																	+				
	VMAPRTLFL		+																					+																	+				

Self	VMAPRTLIL, VMAPRTLLL		+	+										+			+																								+			+	[35]

Self	QMRPVSRVL		+															+	+	+																					+				[45]

Self	SLELGDSAL		+																																						+				[33]
	LLLGPGSGL		+																																						+				

EBV	SQAPLPCVL		+																					+																	+				[34]

EBV	SQAPLPCVL		+	+										+			+																								+			+	[35]

EBV	SQAPLPCVL		+																																									+	[36]

CMV	VMAPRTLIL		+	+									−																												+			+	[37, 39]

CMV	VMAPRTLIL		+						+	+	−	−	−	+				+						+		+		−													+			+	[38]

CMV	VMAPRTLIL, VMAPRTLVL		+																					+																				+	[12]

CMV	VMAPRTLLL	+	+	+					lo	hi	−	−	#	+	−	−		+	+	+					+	+	+														?			+	[41]

CMV	VMAPRTLIL								+			−						+																										+	[40]

HCV	YLLPRRGPRL		+																							+																			[44]

Salmonella	GMQFDRGYL	+	+											−						+						+															+				[43]
	AMLQDIATL	+	+											−						+						+															+				
	KMLRGVNVL	+	+											−						+						+															+				
	VEGEALATL	+	+											−						+						+															+				
	AAVEELKAL	+	+											−						+						+															+				
	AVAKAGKPL	+	+											−						+						+															+				
	KLQERVAKL	+	+											−						+						+															+				

Salmonella	?	+	+						#							−						+				+	+	#					+		+										[46, 49]

Mtb	?		+																							+																			[31, 32]

Mtb	69 peptides	+	+	+	+	+		−										#		+	+					#								*∗*					−		+		+		[30]

Mtb	RMPPLGHEL	+	+	+	+	+	+	+	−		+							#	+	+	+					#	+	#	#	+	#	+				−	+	−	−	#	+	+	+	+	[47]
	VLRPGGHFL	+	+	+	+	+	+	+	−		+							#	+	+	+					#	+	#	#	+	#	+				−	+	−	−	#	+	+	+	+	

Mtb	VMTTVLATL	+	+	+														−		#	−		#			−	+	−	#	−	−	+	−								+	−		+	[48]
	RLPAKAPLL	+	+	+														−		#	−		#			−	+	−	#	−	−	+	−								+	−		+	
	VMATRRNVL	+	+	+														−		#	−		#			−	+	−	#	−	−	+	−								+	−		+	
	RMPPLGHEL	+	+	+														−		#	−		#			−	+	−	#	−	−	+	−								+	−		+	
	VLRPGGHFL	+	+	+														−		#	−		#			−	+	−	#	−	−	+	−								+	−		+	


“+” = positive in most samples; “−” = hardly seen; #: observed in about half of the samples; *∗*: membrane bound TGF-*β*, not secreted.
